# Existing Evidence for the Repurposing of PARP-1 Inhibitors in Rare Demyelinating Diseases

**DOI:** 10.3390/cancers14030687

**Published:** 2022-01-29

**Authors:** Marianna Mekhaeil, Kumlesh Kumar Dev, Melissa Jane Conroy

**Affiliations:** 1 Drug Development Research Group, Department of Physiology, School of Medicine, Trinity College Dublin, D18 DH50 Dublin, Ireland; mekhaeim@tcd.ie (M.M.); devk@tcd.ie (K.K.D.); 2 Cancer Immunology Research Group, Department of Physiology, School of Medicine, Trinity College Dublin, D18 DH50 Dublin, Ireland

**Keywords:** repurposing, PARP-1, leukodystrophies

## Abstract

**Simple Summary:**

Poly (ADP-ribose) polymerase-1 (PARP-1) inhibitors are successful cancer therapeutics that impair DNA repair machinery, leading to an accumulation of DNA damage and consequently cell death. The shared underlying mechanisms driving malignancy and demyelinating disease, together with the success of anticancer drugs as repurposed therapeutics, makes the repurposing of PARP-1 inhibitors for demyelinating diseases a worthy concept to consider. In addition, PARP-1 inhibitors demonstrate notable neuroprotective effects in demyelinating disorders, including multiple sclerosis which is considered the archetypical demyelinating disease.

**Abstract:**

Over the past decade, Poly (ADP-ribose) polymerase-1 (PARP-1) inhibitors have arisen as a novel and promising targeted therapy for breast cancer gene (BRCA)-mutated ovarian and breast cancer patients. Therapies targeting the enzyme, PARP-1, have since established their place as maintenance drugs for cancer. Here, we present existing evidence that implicates PARP-1 as a player in the development and progression of both malignancy and demyelinating disease. These findings, together with the proven clinical efficacy and marketed success of PARP-1 inhibitors in cancer, present the repurposing of these drugs for demyelinating diseases as a desirable therapeutic concept. Indeed, PARP-1 inhibitors are noted to demonstrate neuroprotective effects in demyelinating disorders such as multiple sclerosis and Parkinson’s disease, further supporting the use of these drugs in demyelinating, neuroinflammatory, and neurodegenerative diseases. In this review, we discuss the potential for repurposing PARP-1 inhibitors, with a focus on rare demyelinating diseases. In particular, we address the possible use of PARP-1 inhibitors in examples of rare leukodystrophies, for which there are a paucity of treatment options and an urgent need for novel therapeutic approaches.

## 1. Introduction

Intrinsic DNA repair pathways are employed in response to environmental insults to maintain cellular function and viability [1]. Dysfunctional DNA repair mechanisms lead to a loss of genomic integrity and stability as well as increased risk of errors in the synthesis of RNA and proteins [2]. Altered DNA repair mechanisms are also closely associated with a multitude of cancers [1].

Poly (ADP-ribose) polymerase (PARP) is a family of 17 human enzymes (Table 1) that coordinate DNA repair, transcriptional regulation, cell cycle, oncogene activity, and mitochondrial function, through an ADP-ribosylation post-translational modification named PARylation [3]. This reaction requires the oxidation of nicotinamide adenine dinucleotide (NAD^+^) to synthesise and covalently attach monomers or polymers of ADP ribose to target proteins, which function in the rescue of DNA damage [4].

**Figure 1:**
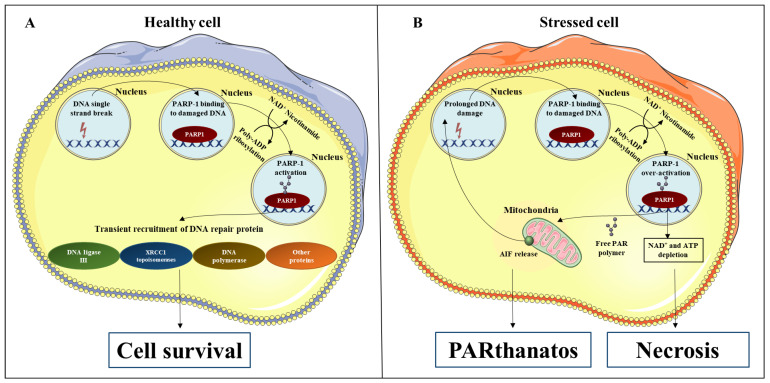
PARP-1 signaling in healthy and stressed cells. (**A**) PARP-1 activation requires NAD^+^ to catalyse the addition of PAR to itself. This same pathway is also used for PARP-1 to add PAR to DNA repair proteins including DNA ligase II, DNA polymerase, and XRCC1 topoisomerases. (**B**) Prolonged DNA damage induces over-activation of PARP-1, leading to NAD^+^ and ATP depletion, which may promote cell necrosis. In addition, some PAR may translocate to the cytosol and bind to mitochondrial receptors, causing the release of AIF, which diffuses to the nucleus and triggers DNA fragmentation. This form of cell death is known as “PARthanatos”. Parts of the figure were generated using images from Servier Medical Art (http://smart.servier.com/ accessed on 15 January 2022).

PARP-1 is the major source of poly-ADP ribose (PAR), accounting for over 90% of PARylation activity [5]. The most well characterized function of PARP-1 is to recognize and repair DNA single-strand breaks (SSBs) by catalysing the addition of PAR to PARP-1 itself and other proteins such as DNA ligase II, DNA polymerase, and XRCC1 topoisomerases (Figure 1A) [6,7]. Such enzymes regulate genomic integrity and play an essential role in the repair of SSBs. The DNA repair process is terminated by the intervention of poly (ADP-ribose) glycohydrolase (PARG) that removes PAR from target proteins [8]. Failure in SSBs repair may progress to double-strand breaks, which are often lethal to cells [9] and are associated with the development and progression of multiple diseases (Table 1). When programmed cell death pathways such as apoptosis occur, PARP-1 is cleaved and inactivated by activated caspases [10]. Deficiencies in PARP-1 inactivation or prolonged DNA damage can result in excessive activation of PARP-1 and consequential NAD^+^ and adenosine triphosphate (ATP) depletion [10,11]. In this case scenario, cells may become necrotic or undergo a less common form of cell death called “*PARthanatos*” [10]. PARthanatos is involved in the pathogenesis of many brain diseases including neurodegeneration and neuroinflammation [10]. This process is triggered by the translocation of free PAR into the cytoplasm and its binding to mitochondrial receptors, resulting in the release of apoptosis-inducing factor 1 (AIF). AIF enters the nucleus and induces DNA fragmentation [10] (Figure 1B).

**Figure 2:**
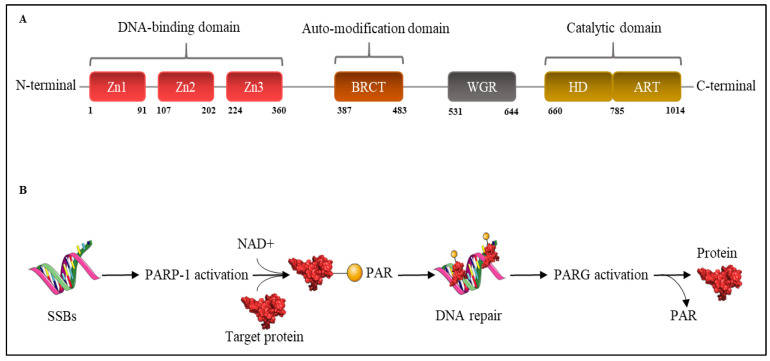
Scheme of PARP-1 structure and mechanism of action. (**A**) PARP-1 is comprised of three main domains starting from N-terminal to C-terminal: DNA binding domain characterized by zinc finger motifs Zn1, Zn2, and Zn3; auto-modification domain with the BRCT motif; WGR domain; catalytic domain formed by HD and ART; (**B**) DNA SSBs are recognized by PARP-1, which become activated and use NAD^+^ to catalyse the addition of PAR to target proteins that contribute to DNA’s reparation. The reaction is ended by PARG’s recruitment that removes PAR from target proteins. Parts of the figure were generated using images from Servier Medical Art (http://smart.servier.com/ accessed on 15 January 2022).

Structurally, PARP-1 has a C-terminal catalytic domain, composed of a helical subdomain (HD) and ADP-ribosyl transferase (ART) fold [13]. Its function is to oxidize NAD^+^ as a substrate to add PAR on target proteins. PARP-1 also has a Trp-Gly-Arg (WGR) domain and an N-terminal DNA-binding domain (DBD) containing three zinc finger motifs (Zn1, Zn2, and Zn3) [13]. Together, these domains allow PARP-1 to detect DNA breaks and physically interact with chromatin [14]. In addition, there is a central auto-modification domain (AMD) constituted by BRCA1 C terminus (BRCT) motif, which acts as the target of covalent auto-modification [14] (Figure 2).

## 2. The Pharmacology of PARP-1 Inhibitors

The chronic activation of PARP-1 is associated with the development of multiple malignancies of the breast, ovarian, uterine, lung, skin, and prostate, along with colorectal cancer, pediatric central nervous system cancer, Non-Hodgkin’s Lymphoma, glioblastoma multiforme, Ewing’s sarcoma, and testicular germ cell tumour [15,16]. Despite the well-known tumourigenic role of PARP-1 that places it as an appealing therapeutic target, the mechanisms underlying its specific function in cancer have not yet been fully elucidated. The most validated hypothesis is that cancer cells and non-cancer cells which are subjected to long-term oxidative stress, extensive DNA damage, or inflammatory conditions, up-regulate PARP-1 to repair mildly damaged DNA and harbour carcinogenetic mutations that promote tumour growth [16]. In addition, PARP-1 strongly contributes to chronic inflammation and genomic instability, two hallmarks of cancer [15]. Furthermore, PARP-1 functions are extended in a disease-specific manner; for example, in breast cancer and prostate cancer, PARP-1 binds to estrogen (ER) and progesterone receptor (PR). respectively, and activates the transcription of ER or PR target genes [17].

At the time of going to press, PARP-1 inhibitors are a targeted therapy for the treatment of breast cancer gene (BRCA)-mutated ovarian and breast cancers [14]. The rational underlying the development of PARP-1 inhibitors is based primarily on the ability of these therapeutics to target cancer cells with a high replication rate and/or a deficiency in DNA repair machinery [14]. In the example of BRCA mutations, these tumour cells harbour unrepaired SSBs, and, in this scenario, PARP-1 activation is fundamental to rescuing DNA damage and cell death. Therefore, PARP-1 inhibition is cytotoxic for cancer cells and is used both as a cancer monotherapy or in combination with chemotherapy [18,19].

The first PARP-1 inhibitor was nicotinamide, a reaction product released from NAD^+^ during the synthesis of PAR [20]. Thereafter, all successive PARP-1 inhibitors have been modelled on nicotinamide and contain a carboxamide group bound to an aromatic ring that competes with NAD^+^ for the catalytic pocket of PARP-1 [20]. As of 2021, US Food and Drug Administration (FDA) and European Medicine Agency (EMA) have approved four PARP-1 inhibitors as anti-cancer therapies for breast or ovarian cancer, namely: Olaparib (IC50 = 13 nM), Rucaparib (IC50 = 80 nM), Niraparib (IC50 = 35 nM), and Talazoparib (IC50 = 3 nM) [20]. These drugs promote cell death in cancer by acting at two levels. Firstly, PARP-1 inhibitors compete with the binding site of NAD^+^ in the catalytic domain of PARP-1 enzymes and inhibit their activity, leading to an increased error in repair of DNA, particularly in rapidly dividing cancer cells [10]. Secondly, PARP-1 inhibitors prevent auto-PARylation and consequently the release of PARP-1, downregulating PARP-1 levels and thus reducing DNA repair in cancer cells [21]. Their high selectivity, oral availability, pharmacokinetic and pharmacodynamic properties, and potency make PARP-1 inhibitors attractive for multiple cancers that share high levels of replication and genomic instability [21]. Although the clinical potential of PARP-1 inhibitors is unquestionable with more than 100 ongoing clinical trials in both solid tumours and hematologic malignancies (clinicaltrials.gov accessed on 11 November 2021), many PARP-1 inhibitors have poor selectivity [22,23]. Their binding affinity for PARP-2 has mostly been reported, due to the high homology of PARP-1 and PARP-2 in the catalytic domain, and their sequence similarity [23]. Therefore, off-target inhibition of PARP-2 is likely to contribute to the efficacy of PARP-1 inhibitors [14]. On the other hand, there is evidence that PARP-2 inhibition could be responsible for adverse reactions such as hematological and gastrointestinal toxicities [24]. As such, there is significant interest in refining the selectivity and potency of PARP-1 inhibitors such as NMS-P118, a selective compound that inhibits PARP-1 80-fold more potently than PARP-2 and totally represses the growth of breast cancer cells and pancreatic ductal adenocarcinoma xenografts [25].

## 3. PARP-1 in Inflammation

**Figure 3:**
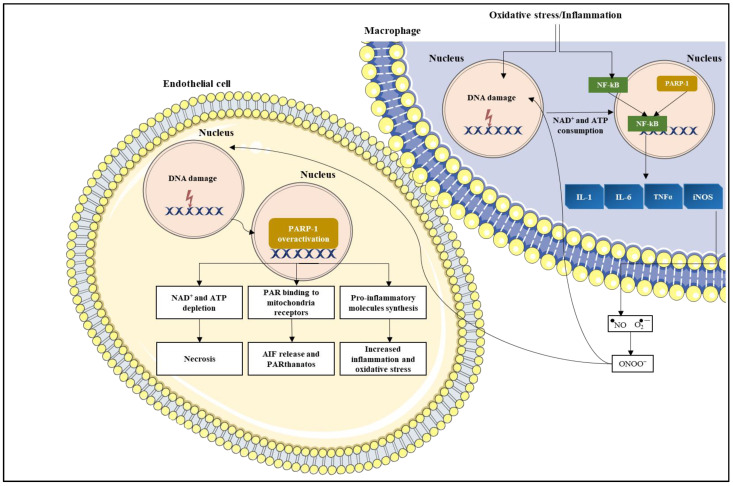
PARP-1 role in inflammation. Macrophages under inflammatory or oxidative stress conditions, activate PARP-1, which in turn positively regulates NF-κB transcription. NF-κB induces the transcription of pro-inflammatory genes including IL-1, IL-6, TNFα, and iNOS. The consequent synthesis of NO that reacts with the superoxide anion to form peroxynitrite (ONOO^−^), causes DNA damage in other cells such as endothelial cells, triggering PARP-1 overactivation. This ultimately leads to cell death through necrosis or AIF release and eventual PARthanatos. Parts of the figure were generated using images from Servier Medical Art (http://smart.servier.com/ accessed on 15 January 2022).

PARP-1 has a key role in chronic inflammation in the context of many inflammatory-driven pathologies as well as in tumours [26]. This has been confirmed by the resistance of PARP-1 knock out (KO) mice or mice treated with PARP-1 inhibitors to various types of inflammation such as lipopolysaccharide-induced septic shock [27,28]. PARP-1′s pro-inflammatory effects are not limited to immune cells and have been observed in astrocytes, endothelial cells, and fibroblasts, contributing to the inflammatory process in nearly all tissues [15,29,30]. PARP-1 achieves this through two key pathways. Firstly, via co-activation of nuclear factor kappa-light-chain-enhancer of activated B cells (NF-κB), which induces the transcription of genes-encoding proteins such as inducible nitric oxide synthase (iNOS), tumour necrosis factor-α (TNFα), cell adhesion molecules (e.g., intercellular adhesion molecule-*1* (ICAM-1), vascular adhesion molecule-*1* (VCAM-1)), interleukin-1 (IL-1), interleukin-6 (IL-6), and pro-metastatic cytokines [31,32]. Together these molecules enhance inflammation, which in turn augments the expression of reactive oxygen species (ROS) and increases genomic instability as well as the sensitivity of surrounding cells to oxidation [26,31]. In addition, NF-κB induces further PARP-1 activation, thus creating a chronic oxidative loop [33]. Secondly, PARP-1 over-activation causes massive PAR synthesis, NAD^+^ and ATP depletion, and, ultimately, cell death [32] (Figure 3). Given the ubiquitous role of PARP-1 in cell physiology, its use as a drug target in other indications outside the field of oncology has been explored and has proved promising. Several FDA-approved PARP-1 inhibitors, particularly Olaparib and Veliparib, have demonstrated cytoprotective and anti-inflammatory effects in preclinical models of non-oncological diseases such as lung inflammatory disorders, neurological disorders (e.g., stroke, Parkinson’s disease, Alzheimer’s disease, multiple sclerosis), diabetes, and myocardial infarction [34,35,36]. PARP-1 inhibitors in chronic inflammatory diseases contribute to reduce oxidative stress, decrease PAR synthesis and consequent AIF release by the mitochondria, prevent NAD^+^ depletion and cell death, decrease NF-κB activation, and reduce expression of adhesion molecules and lymphocyte infiltration [14,15,37]. All these mechanisms can be targeted simultaneously with synergistic effects, thus explaining why lower doses of PARP-1 inhibitors are needed to confer efficacy in non-oncological conditions, compared to cancer [15]. These studies strengthen a case for repurposing these PARP-1 drugs for non-oncological indications, particularly in illnesses for which there are few therapeutic options.

## 4. Role of PARP-1 in the Central Nervous System

PARP-1 is expressed by all brain cells in the central nervous system (CNS) [38]. In addition to its predominant role in the synthesis of PAR, PARP-1 is involved in multiple biological processes in the CNS including cell differentiation and maturation, regulation of cholinergic and glutamatergic signaling, and memory formation [39,40]. Here, we present the current knowledge on the role of PARP-1 in the CNS, its implication in diseases of the CNS, and its potential as a therapeutic target in neurological inflammatory and demyelinating diseases.

### 4.1. PARP-1 Expression in Neurons

Neurons are highly susceptible to free radical molecules, which are generated during cell respiration, oxidative stress, and neurotransmission, and can lead to DNA damage as well as PARP-1 activation [41]. Under basal conditions, PARP-1 is associated with signaling pathways, which are essential for neuronal survival and synaptic plasticity such as Akt, extracellular signal-regulated kinases (ERKs), and the transcription factor cyclic AMP response element-binding protein (CREB) [42].

In acute neuronal injury, PARP-1 activation is thought to induce neuronal death via AIF translocation from mitochondria to the nucleus [43]. It has been described in both human and animal cells that NMDA receptor-mediated excitotoxicity is induced by the synthesis of nitric oxide (NO), which reacts with the superoxide anion to form peroxynitrite (ONOO^−^), a potent toxic molecule that causes high levels of neuronal DNA damage and leads to PARP-1 activation [44,45]. Subsequently, treatment of neuronal cells with PARP-1 inhibitors (Rucaparib, Veliparib, Talazoparib, Olaparib, and DPQ) can rescue cells from oxidative stress or NMDA-induced cell death, indicating potential use of these drugs in neuronal diseases [43,45].

PARP-1 chronic activation, which occurs in many CNS disorders, not only causes neuronal death via apoptotic-independent pathways but is also implicated in axonal degeneration [46]. The mechanisms underlying this toxicity include the depletion of NAD^+^ and ATP, which are crucial biomolecules for neuronal viability, growth, axonal survival, and motility [46]. In addition, PARP-1 is responsible for cytotoxic expression of glutamate-type Ca^2+^-permeable AMPA receptors in hippocampal neurons and therefore neuronal death [47,48]. Overall, PARP-1 inhibition is a promising therapeutic strategy to rescue neuronal loss and promote axonal regeneration in chronic brain injury [49,50].

### 4.2. PARP-1 Expression in Oligodendrocytes

Oligodendrocytes represent 5–10% of the glial cell population [51] and are responsible for axonal myelination, a process that is fundamental for axonal integrity and rapid neuron communication [52]. Oligodendrocytes differentiate from oligodendrocyte progenitor cells (OPCs) into immature and then mature myelinating cells, before and after birth in a highly regulated manner [53]. All cell linages of oligodendrocytes (OPCs, immature and mature myelinating cells) are susceptible to oxidative stress, inflammation, and DNA damage [54]. Damage to these cells ultimately results in loss of myelin around neuronal axons and in synaptic conduction aberrancies, axonal dysfunction, and neurological disorders such as multiple sclerosis and leukodystrophy [54].

During CNS development, PARP-1 transcript concentration is increased in oligodendrocytes, where it is thought to act as a positive regulator of oligodendrocyte differentiation and maturation [54]. Recent publications have demonstrated that PARP-1 KO mice present a significant reduction in oligodendrocyte density and essential myelin proteins (e.g., myelin oligodendrocyte glycoprotein (MOG), myelin basic protein (MBP), myelin associated glycoprotein (MAG)) with consequential hypomyelination in the corpus callosum [55]. In addition, purified primary OPCs from PARP-1 KO mice show severe inhibition of differentiation and maturation [55]. These studies suggest that therapeutic enhancement of PARP-1 could be a viable strategy to induce remyelination in demyelinating diseases. There are, however, contrasting reports in the literature showing that PARP-1 inhibition by 4-hydroxyquinazoline (4HQ) prevents oligodendrocyte death in diseases such as multiple sclerosis and ischemia [56,57]. Additional studies have reported that the role of PARP-1 in oligodendrocytes may depend on cellular age and a specific stage of cell differentiation [58]. Despite differences reported, these studies support the hypothesis that PARP-1 plays a direct role in oligodendrocyte differentiation, viability, and myelination state.

### 4.3. PARP-1 Expression in Microglia

Microglia constitute 10–15% of glial cells in the CNS and are the primary line of immune defense in the brain [59]. Specifically, microglia are considered the resident immune macrophages of the CNS, with an ability to phagocytose pathogens, dead cells, protein aggregates, and soluble antigens that might prove harmful to the CNS [60]. Furthermore, microglia are pivotal mediators of neuroinflammation and the main producer of proinflammatory cytokines and chemokines in the CNS [61]. Neuroinflammatory disorders are often associated with an excessive accumulation of reactive microglia, which are seen to contribute to neuronal damage in neurodegenerative diseases, stroke, brain injury, brain tumours and infections, and other brain diseases [62,63]. Therefore, therapeutically targeting reactive microglia and their inflammatory responses could be clinically useful.

Importantly, the interaction between PARP-1 and NF-κB is required for microglial migration, activation, proliferation, and production of pro-inflammatory molecules such as iNOS, TNF-α, ICAM-1, CCL2, as well as ROS [64]. Furthermore, PARP-1 deletion or inhibition in microglia promotes neuroprotection in the injured brain [65]. This was evident in excitotoxic damaged organotypic hippocampal slice cultures derived from mice, in which PARP-1 downregulation inhibited CD11a expression, a crucial microglial integrin, and attenuated microglial migration towards the sites of neuronal injury, thus rescuing neuronal damage [66]. In addition, inhibition of microglia PARP-1 by the selective inhibitor, PJ34, has been shown to reduce the production of pro-inflammatory molecules like ICAM-1, granulocyte-macrophage colony-stimulating factor (GM-SCF), and CXCL9 [67].

### 4.4. PARP-1 Expression in Astrocytes

Astrocytes are the most abundant glial cells accounting for 25% of total brain cells [68]. Along with their traditional function of regulating CNS homeostasis, astrocytes participate in innate immune responses and neuroinflammation, mitochondrial biogenesis, synapse modulation, neuronal survival, defense from oxidative stress, and elimination of protein aggregates [68,69]. Astrocytes play an important role in supporting neurons, forming a functional tripartite synapse composed of two neurons and one astrocyte [70]. In this unit, neurons and astrocytes collectively modulate synaptic behaviour by neurotransmitter and gliotransmitter-based crosstalk [71]. In addition, astrocytes support CNS myelination by producing the major fraction of brain cholesterol, where an absence in astrocyte cholesterol production results in hypomyelination [72]. Furthermore, astrocytic endfeet that express the water channel aquaporin 4 (AQP4) and the Kir4.1 K^+^ channel, are one of the major regulators of blood–brain barrier (BBB) integrity [73]. Overall, astrocytes play a crucial and multifaceted role in regulating brain homeostasis and in maintaining brain health.

Similar to microglia, PARP-1 is crucial for astrocyte activation and pro-inflammatory cytokine production [74]. PARP-1 is constitutively expressed in the nucleus of astrocytes [75]. Primary astrocytes derived from PARP-1 KO mice exhibit partial suppression of NO, interleukin-1β (IL-1β), TNF-α, and CCL2 production compared to wild type (WT) cells [75]. Excessive activation of PARP-1 leads to astrocyte cell death via NAD^+^ and ATP depletion. In addition, dysfunction of PARP-1 alters the permeability of the inner mitochondrial membrane, causing the release of apoptotic factors into the cytoplasm [76]. PARP-1 also regulates astrocytic glutamate transporters, which are essential in glutamate neurotransmission and neuroprotection [77]. The activation of PARP-1 with the DNA alkylating agent, *N*-methyl-*N*′-nitro-*N*-nitrosoguanidine (MNNG), in astrocytes causes significant reduction in astrocytic glutamate uptake, which is rescued by treatment with PARP-1 inhibitor PJ34 [77]. In contrast, however, studies have reported that Talazoparib and Olaparib worsen the astrocytic toxic effect caused by sodium nitroprusside [78]. Despite these conflicting studies, there is growing interest in targeting microglial and astrocytic PARP-1 as a novel potent anti-inflammatory and neuroprotective therapy for chronic neuroinflammatory diseases.

### 4.5. PARP-1 Inhibitors for Use in Brain Cancer

Glioblastoma multiforme (GBM) is the most common and aggressive form of brain cancer in adults [79]. Current treatments, such as surgical resection, have limitations due to the infiltrative nature of GBM and the lack of identifiable tumour margins [80]. Treatment with alkylating agents such as temozolomide (TMZ) is challenged by the blood–brain barrier (BBB), with only ~20% of systemically administered drug reaching the tumour [81]. Chemotherapy efficacy is also significantly hampered by treatment resistance, resulting in GBM regrowth [81]. The BBB has always represented a major obstacle to the delivery of drugs to GBM tumours, and PARP-1 inhibitors have shown some success in clinical studies in overcoming this [82]. With a better understanding of GBM biology, PARP-1 has emerged as a key player in the pathology of this malignancy and has been shown to be overexpressed and inversely correlated with survival in GBM patients [83]. PARP-1 inhibition is currently under investigation to evaluate its efficacy to sensitize GBM tumours to chemotherapy and radiotherapy (Table 2). Among FDA-approved PARP-1 inhibitors, Talazoparib and Rucaparib reported a poor distribution and a limited TMZ sensitization in an orthotopically implanted GBM patient-derived xenograft model [84,85]. In contrast, Veliparib shows more promise, demonstrating superior brain penetrance and effective TMZ sensitisation [86,87]. In addition, Olaparib, which has proved a discrete BBB penetration in patient-derived xenografts model [85], is currently in phase II studies as a single agent therapeutic for patients with advanced glioma, cholangiocarcinoma, or solid tumours with isocitrate dehydrogenase 1 or 2 (IDH1 or IDH2) mutations (Table 2).

Overall, the overexpression of PARP-1 in a multitude of brain diseases and the capacity of PARP-1 inhibitors to penetrate the BBB, places this enzyme as a desirable therapeutic target for brain disorders that share chronic inflammation and/or oxidative stress and a high rate of DNA damage. In the next sections we will review the role of PARP-1 in examples of neurodegenerative diseases.

## 5. Evidence for PARP-1 as a Drug Target for Neurodegenerative Diseases

**Figure 4:**
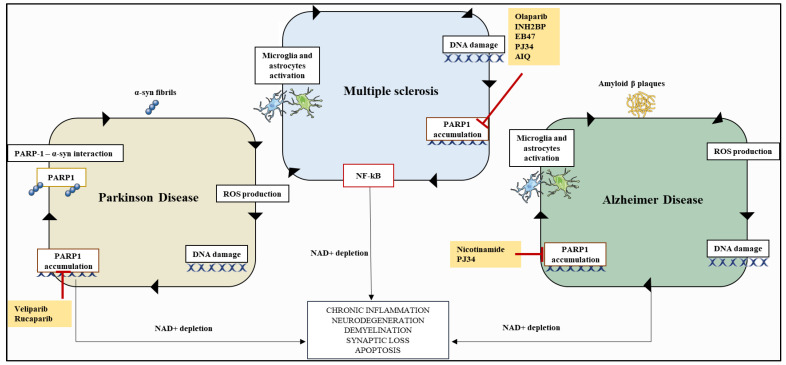
Overactivation and pre-clinical evidence of PARP-1 inhibitor efficacy for PD, AD, and MS. Fibrillary α-syn in PD causes PAR accumulation, a marker of PARP-1 activation, in neurons in the substantia nigra and in the striatum, which subsequently interacts with α-syn and accelerates its fibrillization. PARP-1 selective inhibition with Rucaparib or Veliparib, reduced α-syn aggregation, spreading, and neurotoxicity in a PD mouse model. In MS, PAR accumulation is measured in cells surrounding demyelinated plaques. PARP-1 inhibition with selective inhibitors PJ34 or INH2BP in the EAE model markedly reduces neuro-inflammation by preventing NF-κB, microglia, and astrocytes over-activation. In AD, amyloid β plaques enhance ROS production, leading to PARP-1 over-activation which, in different transgenic mouse models, is rescued by the treatment with nicotinamide or PJ34. Parts of the figure were generated using images from Servier Medical Art (http://smart.servier.com/ accessed on 15 January 2022).

Chronic inflammation and DNA damage underpin several neurological disorders, including Alzheimer’s disease, Parkinson’s disease, and multiple sclerosis [88]. Multiple sclerosis is an autoimmune, inflammatory, demyelinating disease of the CNS [89]. In the rodent model of multiple sclerosis, experimental allergic encephalomyelitis (EAE), PAR, the marker of PARP-1 activation, accumulates in astrocytes as well as oligodendrocytes, microglia, and neurons, surrounding demyelinated plaques [90]. PARP-1 inhibition with selective inhibitors (PJ34 or INH2BP) in the EAE model reduces disease progression and improves symptoms, via a reduction in the expression of inflammatory cytokines TNF-α, IL-1β, interferon-γ (IFN-γ), interleukin-2 (IL-2), and iNOS in the spinal cord [91,92] (Table 3). In addition, PARP-1 inhibitors reduce infiltration of immune cells via alterations of the BBB and/or via inhibition of PARP-1 activity in monocytes [93]. Lack of immune cell transmigration across the BBB is also likely to be associated with a decrease in immune cell adhesion to brain endothelium due to altered conformation of integrins such as VLA-4 and LFA-1 [92,94,95]. Parkinson’s disease is caused by an accumulation of fibrillary α-synuclein (α-syn) and is associated with elevated levels of oxidative stress and likely the activation of PARP-1 [96]. The injection of preformed α-syn fibrils into mouse brain results in the accumulation of PAR, likely via enhanced PARP-1 enzyme activity, in neurons of the substantia nigra and striatum [88,97]. This accumulated PAR subsequently interacts with α-syn, resulting in a positive loop that accelerates its fibrillization. Genetic deletion of PARP-1 or treatment with Rucaparib or Veliparib reduces α-syn aggregation, spreading, and neurotoxicity in primary neuron cells [97] (Table 3). Several studies have also demonstrated that PARP-1 is over-activated in brains of patients with Alzheimer’s disease [98,99]. Furthermore, PARP-1 inhibition using nicotinamide or PJ34, which have now been established as weak inhibitors [100], in transgenic mouse models of Alzheimer’s disease ameliorates brain pathology through a reduction of β-amyloid production [101] (Table 3). Taken together, these pre-clinical reports provide strong evidence for the utility of PARP-1 inhibitors in neurological disorders, neuroinflammation, and neurodegenerative diseases (Figure 4).

## 6. Potential Utility of PARP-1 Inhibitors in Leukodystrophies

### 6.1. The Clinical Challenge and Therapeutic Landscape of Leukodystrophies

Leukodystrophies are a group of heterogeneous, genetically-based disorders affecting the development or maintenance of the myelin in the white matter of the CNS [112]. Collectively, leukodystrophies have an incidence of 1 in 7700 live births and can present at any age from infancy to adulthood, with a considerable variability in disease progression and clinical presentation [113]. Patients with leukodystrophies experience a large array of significant and disabling symptoms including motor impairment, dysautonomia, cognitive impairment, and ataxia [113]. Most of the diagnosis for leukodystrophies are not precise and are based on a combination of history, expected prevalence, physical and neurologic features, and radiological examination [114]. More promisingly, recent advances in genetic medicine and imaging have led to the identification of specific genetic and biochemical defects associated with individual leukodystrophies including metachromatic leukodystrophy and X-linked adrenoleukodystrophy, which are the most frequent diagnosed among leukodystrophies, and Krabbe disease [115].

In the past decades, existing dogma has been questioned suggesting that mutations within myelin- or oligodendrocyte-specific genes were the sole causative factors behind leukodystrophies [116]. Nowadays, leukodystrophies are linked to defects in astrocytes, microglia, axons, and blood vessel function [116]. Causes of leukodystrophies have also been associated with pathological mechanisms that share features with the archetypical demyelinating disease, multiple sclerosis, including BBB disruption, disorders of DNA transcription, translation, production of essential proteins for myelin, and neuroinflammation [117]. Both diagnosis and treatment of leukodystrophies possess a significant challenge due to the limited information regarding the mechanisms behind their pathologies. Symptomatic treatments can decrease the burden of events and assist somewhat in the quality of life [115]. To date, however, effective cures for patients with leukodystrophies are greatly lacking. In this scenario, reducing neuroinflammation, which plays a pivotal role in leukodystrophy progression presents a desirable strategy, either possibly as a monotherapy or part of a combinatorial approach. The repurposing of approved drugs with a clinically proven ability to attenuate inflammation, could be an attractive strategy to pursue. Below we address the role of PARP-1 in exemplar leukodystrophy diseases and discuss the possibility of repurposing PARP-1 inhibitors for these diseases.

### 6.2. Krabbe Disease—Psychosine as a Toxin

Globoid cell leukodystrophy (Krabbe disease) is a rare autosomal recessive neurodegenerative disorder that affects approximately 1/100,000 live births [118]. Krabbe disease typically has an early onset, is rapidly progressing, and is inevitably fatal in infants [119]. The majority (85–90%) of cases are of the infantile form and are characterized by symptoms including irritability, hypersensitivity, psychomotor arrest, and hypertonia, followed by optic atrophy, rapid mental and motor deterioration, and seizures [118]. Typically, death usually ensues within the first 2 years of life [120,121]. Krabbe disease is caused by a mutation in the lysosomal enzyme galactosylceramidase (GALC) [122], resulting in the accumulation of a toxic lipid metabolite psychosine (galactosylsphingosine) and, to a lesser extent, β-galactosylceramide [123]. Progressive accumulation of psychosine in the brains of Krabbe disease patients is thought to be the major driver of this illness [120]. Pathological features of Krabbe disease include profound demyelination and almost complete loss of oligodendrocytes in the white matter, reactive astrocytosis, and infiltration of numerous multinucleated macrophages termed “globoid cells” [120]. Several reports have demonstrated that the cellular cytotoxicity caused by psychosine is in part mediated by inflammatory molecules, such as NF-κB, AMP-activated protein kinase (AMPK), prostaglandin D, iNOS, and pro-inflammatory cytokines among all TNF-α and IL-6 [124]. There is currently no cure for KD, except for hematopoietic stem cell transplantation, which is effective only if performed at the early stage of the disease [125]. More recently, we demonstrated the potential therapeutic avenue utilizing the oral multiple sclerosis drug, fingolimod, in the Twitcher mouse model of Krabbe disease [126].

### 6.3. X-Linked Adrenoleukodystrophy—The Build-Up of Very Long Fatty Acids

X-linked adrenoleukodystrophy (X-ALD) is a severe neurodegenerative disorder with an overall incidence of 1/17,000 [127]. X-ALD is characterized by progressive demyelination within the central and peripheral nervous system, adrenal insufficiency, and accumulation of very-long-chain fatty acids (VLCFA) in plasma, skin fibroblasts, and all tissues [127]. The phenotype of this disease depends on the age of presentation and the organs affected [128]. The hallmark feature that characterizes childhood cerebral X-ALD is developmental regression, followed by severe disability, coma, and death [129]. The adrenal gland dysfunction characterizes a phenotype named Addison disease that develops usually during adolescence [128]. In the third decade of life, the typical symptoms are walking difficulties, unbalanced gait, and bowel/bladder sphincter dysfunction [130]. The toxic effects of VLCFA accumulation in X-ALD are still not fully understood, although most of the symptoms are associated with inflammation and oxidative stress [131]. The mechanisms that lead to the inflammatory reaction in cerebral X-ALD might involve abnormal acylation of gangliosides and phospholipids by VLCFA [128]. The abnormal accumulation of lipids results in immune reaction of brain macrophages and astrocytes [132]. These changes usually result in the loss of the myelin sheets, oligodendrocytes, and neuronal axons [128]. In case of X-ALD, treatment with monoenoic fatty acids reduces inflammation [133]. The inhibition of iNOS by l-*N*-methylarginine normalizes VLCFA levels and β-oxidation in cytokine-treated cell model [134]. At present, there is no effective treatment for most of the forms of X-ALD. Supportive care by optimizing nutrition, occupational therapy, and respiratory support can help alleviate some of the symptomology but typically does not significantly impact survival or long-term outcomes [133]. Corticosteroid and mineralocorticoid replacement therapy is the recommended therapy in those with impaired adrenal gland function [133]. Some reports have shown that allogeneic hematopoietic cell transplant (HCT) may have beneficial effects in X-ALD asymptomatic patients at the time of diagnosis or those with mild symptoms and CNS involvement [127].

### 6.4. Metachromatic Leukodystrophy—Sulfatides as the Culprits

Metachromatic leukodystrophy (MLD) is a demyelinating, autosomal recessive genetic leukodystrophy with an estimated birth prevalence of approximately 1–2/100,000, and an incidence of 1/40,000 births [135]. MLD is a progressive disease that causes the loss of muscle and cognitive function, as well as progressive loss of vision [136,137]. Lifespan often depends on the age of diagnosis. In the late infantile form of this disease, death typically occurs within 5–6 years, while juvenile and adult form may facilitate survival until early adulthood or more [136]. The disease is caused by mutations in the arylsulftase A gene (ARSA) and consequentially a deficient activity of the arylsulfatase A lysosomal enzyme [138]. The loss of function in ARSA enzyme activity leads to the accumulation of sulfatides in oligodendrocytes, which result in the dysfunction and destruction of the neuronal myelin sheaths [138]. Sulfatides also accumulate in visceral organs, pancreas, liver, adrenal glands, lymph nodes, and ovaries [138]. Inflammation is a key driver of MLD disease progression, with elevated levels of CCL2, interleukin-1 receptor antagonist (IL-1Ra), interleukin 8 (IL-8), and macrophage inflammatory protein 1β (MIP-1β) in the cerebral spinal fluid of patients with MLD [139]. Furthermore, excessive glial cell proliferation and microglia hyperactivation are observed in a mouse model of MLD [140]. Microglia markers in human autopsy of MLD and adrenoleukodystrophy reveal that microglial death is caused by DNA fragmentation and precedes demyelination [141]. Moreover, in mouse models of multiple sulfatase deficiency, sulfatide accumulation induces mRNA expression of TNF-α among all other pro-inflammatory cytokines, and T-cell infiltration [142]. Various therapeutic approaches to MLD have been tested in experimental animal models, but so far, no curative treatment is available. Among the promising approaches with potential clinical utility are: (i) enzyme-replacement therapy (ERT); (ii) bone marrow transplant (BMT); (iii) gene therapy by ex vivo transplantation of genetically modified hematopoietic stem cells (HSC); and (iv) AAV-mediated gene therapy directly to the CNS [138].

## 7. Future Potential for Targeting PARP-1 in Leukodystrophies

To date, there is no direct evidence of PARP-1 involvement in leukodystrophies such as KD, X-ALD, or MLD, and as of yet, there are no PARP-1 inhibitors under clinical investigation in this disease space. There are, however, several hypothetical and mechanistic links that are worthy of mention. First, leukodystrophies such as KD, X-ALD, or MLD are associated with altered levels of inflammatory signals such as NF-κB and TNF-α [127,136,142]. These inflammatory intracellular and extracellular signaling molecules are well known to be regulated by PARP-1. Second, the neurotransmitter and neuromodulator NO (synthesized by iNOS) is overproduced in glial cells of demyelinating diseases including X-ALD and KD [143,144]. It is noteworthy that NO is partially suppressed in PARP-1 KO mice, indicating that PARP-1 inhibitors may control levels of NO in these diseases. Third, DNA fragmentation is augmented in leukodystrophies. For example, in cultured fibroblasts and glia-derived MOCH-1 cells with characteristics of myelinating cells, psychosine causes cytotoxic cell death and DNA fragmentation [145]. In this case, targeting PARP-1 may provide benefits in controlling DNA fragmentation. Finally, PARP-1 plays a pivotal role in disrupted BBB, which is observed in leukodystrophies [117]. PARP-1 inhibition with 5-aminoisoquinoline or Olaparib in primary human brain microvascular endothelial cells (BMVEC) stimulated with TNF-*α*, increases expression of tight junctions, augments BBB integrity, and decreases human monocyte adhesion and migration through the BBB. PARP-1 inhibitors also down-regulate expression of inflammatory genes and secretion of pro-inflammatory factors increased by TNF-*α* in BMVEC [95]. Overall, the current data indicate that PARP-1 might play a role in the mechanisms underlying leukodystrophies such as KD, X-ALD, or MLD based on the effects of PARP-1 inhibition on inflammatory and neuromodulatory mediators. Further interrogation of PARP-1 expression and its potential dysregulation is warranted before it can be considered as a potential therapeutic target for these disorders.

## 8. Considerations for Long-Term Use of PARP-1 Inhibitors in Leukodystrophies

The clinical benefit of PARP-1 inhibitors outside the oncological disease space raises questions about potential therapeutic challenges and side effects of long-term treatment. One of the most common objections against the use of PARP-1 inhibitors for chronic diseases relates to the potential increased risk of mutagenesis or oncogenesis. Indeed, inhibition of PARP-1 or gene disruption augments the frequency of chromosomal aberrations after treatment with genotoxic agents [146,147]. However, the increased susceptibility to genotoxic agent-induced genomic instability does not always result in the insurgence of spontaneous tumours. Multiple animal studies have shown that treatment with PARP-1 inhibitors for extended time periods or the deletion of PARP-1 gene, does not lead to any predisposition to tumourigenesis [34,148]. Moreover, potent and specific PARP-1 inhibitors such as GPI 6150 do not induce genetic instability compared to less specific compounds like 3-aminobenzamide [149,150].

In addressing the caution regarding the risk of tumourigenesis by PARP-1 inhibition, it is important to consider that PARP-1 has a key role in oxidative stress and inflammation. In chronic inflammatory-driven pathologies in which DNA damage is induced by the disease itself, PARP-1 inhibitors may reduce the release of mitochondrial cell death factors such as AIF, and downregulate NF-κB and pro-inflammatory cytokines transcription, ultimately decreasing NO synthesis and consequent peroxynitrite-induced DNA damage.

The multifaceted role of PARP-1 together with the insufficient data in the literature that report mostly preclinical studies on short duration of treatments, suggest that further disease-specific studies are warranted to ascertain the risk:benefit ratio pertaining to the use of PARP-1 inhibitors in chronic disease.

Considering that for many chronic inflammatory diseases there are no curative treatments, the use of PARP-1 inhibitors could be a valuable therapeutic strategy. However, not all chronic indications are equal. Several severe and debilitating chronic indications with no alternative therapeutic options (e.g., leukodystrophies or Parkinson’s disease) represent a significant unmet clinical need and therefore remain candidates for urgent drug repurposing. In these indications, the currently available therapeutic options are extremely limited and of marginal efficacy. For indications of this type, treatment with PARP-1 inhibitors may be considered in an intermittent fashion. Alternatively, low dose PARP-1 inhibition may be investigated for its clinical efficacy.

## 9. Concluding Remarks

Many neurological disorders lack specific therapeutic candidates due to the limited understanding of molecular mechanisms underlying their pathology and potential drug targets that underpin such diseases. PARP-1 is an essential player in DNA integrity maintenance, cellular energy metabolism, and inflammation. Therefore, it is not surprising that dysregulation of PARP-1 is associated with chronic inflammatory diseases as well as cancers. Inhibition of PARP-1 in cancer has led to the marketing of four PARP-1 inhibitors for breast cancer and ovarian cancer, namely, Olaparib, Rucaparib, Niraparib, and Talazoparib. PARP-1 inhibitors could also be used in the therapy of inflammatory diseases, including neurological disorders, as inhibition of ADP-ribosylation activity lessens neurodegeneration and demyelination in several animal disease models (e.g., Parkinson’s disease, Alzheimer’s disease, and multiple sclerosis). These data lead us to hypothesize on putative mechanisms linked to PARP-1 signaling that are aberrant in three exemplar leukodystrophy diseases associated with demyelination: Krabbe disease, X-linked adrenoleukodystrophy, and Metachromatic leukodystrophy, for which no therapeutic options currently exist. Targeted drugs with potential to penetrate the BBB and attenuate neuroinflammation are highly desirable for these poor prognosis diseases. Therefore, here we propose that PARP-1 inhibitors are worthy candidates for repurposing for such rare diseases, either as monotherapies or as combination therapies. Further insights into the function of PARP-1 in brain homeostasis and dysregulation in pathological states may assist in the development of PARP-1 inhibitors for rare demyelinating diseases such as leukodystrophies.

## Figures and Tables

**Table 1:** Classification of PARP enzymes and associated pathologies. The PARP family includes 17 enzymes in humans. Most PARPs are localized in nucleus and cytoplasm and contribute to genome integrity, metabolic regulation, and immune response. Mutations or dysfunction in PARP activity are associated with distinct pathologies. Sources: Genecards.org; Proeinatlas.org; Luscher B et al., 2021 [12].

PARP Enzyme	Function	Localization	Catalysed Reaction	Associated Pathology
PARP-1	DNA break repair, chromatin regulation and transcription, cell cycle, metabolic regulation, inflammation	Nucleus	Poly-ADP-ribosylation	Most malignancies, neurodegenerative diseases, brain injury, inflammatory-based diseases, metabolic disorders, Diphtheria, Xeroderma Pigmentosum, Complementation Group A
PARP-2	DNA break repair, chromatin regulation and transcription, cell cycle, metabolic regulation, inflammation	Nucleus, Cytoplasm	Poly-ADP-ribosylation	Most malignancies, neuroinflammation, brain injury, metabolic disorders, Osebold-Remondini Syndrome, Cockayne Syndrome
PARP-3	DNA break repair, chromatin regulation and transcription, cell cycle	Nucleus, Cytoplasm	Mono-ADP-ribosylation	Osebold-Remondini Syndrome, Arthrogryposis, Renal Dysfunction, Cholestasis 1
PARP-4	Tumorigenesis, immune response	Nucleus, Cytoplasm	Mono-ADP-ribosylation	Osebold-Remondini Syndrome, primary thyroid, breast cancer
TNKS1	Regulation of telomeres and mitosis, inflammation, metabolic regulation, stress response	Cytoplasm	Poly-ADP-ribosylation	Cherubism, Lung Acinar Adenocarcinoma, Herpes simplex and Epstein Barr viral infections, severe obesity
TNKS2	Regulation of telomeres and mitosis, inflammation, metabolic regulation	Cytoplasm	Poly-ADP-ribosylation	Cancer, Cherubism, systemic sclerosis, severe obesity, Arthrogryposis, Renal Dysfunction, Cholestasis 1
PARP-6	DNA break repair, tumour suppressor, dendrite morphogenesis in neuron	Cytoplasm	Mono-ADP-ribosylation	Microencephaly, Intellectual disabilities, Epilepsy, Renal cancer, Cervical cancer, Colorectal cancer, Porokeratosis
PARP-7	DNA break repair, chromatin regulation and transcription, innate immune response, neuronal function	Nucleus, Cytoplasm	Mono-ADP-ribosylation	Retinitis Pigmentosa, Breast cancer
PARP-8	Assembly or maintenance of membranous organelles	Cytoplasm	Mono-ADP-ribosylation	Developmental And Epileptic Encephalopathy, Arthrogryposis, Renal Dysfunction, Cholestasis
PARP-9	DNA break repair, chromatin regulation and transcription, immune response, cell migration	Nucleus, Cytoplasm	Mono-ADP-ribosylation	B-Cell Lymphoma, Lymphoma
PARP-10	DNA break repair, chromatin regulation and transcription, RNA processing, immune response	Cytoplasm, Golgi apparatus	Mono-ADP-ribosylation	Diphtheria, Arthrogryposis, Renal Dysfunction, Cholestasis 1
PARP-11	DNA break repair, chromatin regulation and transcription, nuclear envelope stability, immune response	Nucleus, Cytoplasm	Mono-ADP-ribosylation	Osteogenesis Imperfecta, Arthrogryposis, Renal Dysfunction, Cholestasis 1
PARP-12	DNA break repair, chromatin regulation and transcription, RNA processing, stress response, immune response	Cytoplasm	Mono-ADP-ribosylation	Renal and liver cancer, Osebold-Remondini Syndrome, Osteogenesis Imperfecta
PARP-13	Anti-viral factor, chromatin regulation and transcription, stress response, RNA processing, immune response	Cytoplasm	Inactive	Viral infections, Cancer
PARP-14	DNA break repair, chromatin regulation and transcription, immune response, inflammation, metabolic regulation, stress response	Nucleus, Cytoplasm	Mono-ADP-ribosylation	Cancer, atherosclerosis, allergic inflammation
PARP-15	DNA break repair, chromatin regulation and transcription, RNA processing, stress response	Mitochondria	Mono-ADP-ribosylation	Arthrogryposis, Renal Dysfunction, Cholestasis 1, Bejel
PARP-16	Activate unfolded protein response (UPR) effectors in the endoplasmic reticulum	Cytoplasm	Mono-ADP-ribosylation	Osebold-Remondini Syndrome, Arthrogryposis, Renal Dysfunction, Cholestasis 1

**Table 2:** Clinical trials on PARP inhibitors in brain tumours. Summary table highlights the current PARP inhibitors in clinical trials for brain tumours.

PARP Inhibitor	Clinical Trials	Indication	ClinicalTrials.gov Identifier
Olaparib	Phase I	In combination with Temozolomide for the treatment of patients with relapsed glioblastoma	NCT01390571
	Phase II	Treatment of patients with advanced glioma, cholangiocarcinoma, or solid tumours with IDH1 or IDH2 mutations	NCT03212274
NMS-03305293	Phase I	In combination with Temozolomide for the treatment of patients with diffuse gliomas	NCT04910022
	Phase II	In combination with Temozolomide for the treatment of patients with IDH wild type recurrent glioblastoma	
Fluzoparil	Phase II	In combination with Temozolomide for the treatment of patients with recurrent glioblastoma	NCT04552977
BGB-290	Phase I/II	In combination with Temozolomide for the treatment of patients with recurrent gliomas with IDH1/2 mutations	NCT03914742
Veliparib	Phase I	In combination with temozolomide work for the treatment of children with recurrent/refractory CNS tumours	NCT00994071
	Phase I	In combination with whole brain radiation therapy for the treatment of cancer patients with brain metastases	NCT00649207
	Phase II	In combination with radiation therapy, and temozolomide for the treatment of patients with newly diagnosed malignant glioma without H3 K27M or BRAFV600 mutations	NCT03581292
	Phase II	In combination with radiation therapy, and temozolomide for the treatment of patients with newly diagnosed diffuse pontine gliomas	NCT01514201
	Phase II	Cisplatin with or without Veliparib for the treatment of patients with recurrent or metastatic triple-negative and/or BRCA mutation-associated breast cancer with or without brain metastases	NCT02595905
Talazoparib	Phase II	In combination with Carboplatin for the treatment of patients with recurrent high-grade glioma	NCT04740190
Niraparib	Early Phase I	Treatment of patients with newly diagnosed glioblastoma and recurrent glioma	NCT05076513
	Phase II	Treatment of patients with recurrent glioblastoma	NCT04221503

**Table 3:** Pre-clinical studies of PARP-1 inhibitors in neurodegenerative diseases. Table displaying examples of in vitro and in vivo models of brain disorders in which PARP-1 inhibitors have shown efficacy.

Disease/Unhealthy Condition	In Vivo Pre-Clinical Model	Effects of PARP-1 Inhibitor	In Vitro Pre-Clinical Model	Effect of PARP-1 Inhibitor	References
Parkinson’s disease	MTPT mouse model	Benzamide show protective effects against the catecholamine depletions induced by MPTP in cortex and striatum and prevents NAD^+^ and ATP depletion.	Neurons purified from a Parkinson’s disease mouse model injected with preformed α-synuclein fibrils	Rucaparib or Veliparib reduces α-synuclein phosphorylation and aggregation, spreading and neurotoxicity	[97,102,103]
Alzheimer’s disease	3 × Tg-AD mouse model	Nicotinamide reduces levels of phosphorylated species of tau and β-amyloid in the hippocampus and cerebral cortex and restores cognitive functions.	Rat pheochromocytoma (PC12) cells treated with 1 μM Aβ 1–42 oligomers Neuroblastoma cells (SH-SY5Y) treated with Aβ_25–35_ fragment	PJ-34 enhances transcription of antioxidant genes and regulation of mitochondria function. MC2050 diminishes NF-κB activation, ROS production, and pro-apoptotic proteins.	[101,104,105,106]
Stroke	Mouse model of middle cerebral artery occlusion (MCAO) Mouse model of transient middle cerebral artery occlusion MCAO rat model injected with thrombin	PJ34 alleviates post-stroke neuro-inflammation and neurological deficits and suppresses microglial activation. Olaparib reduces infarct size, immunoglobulin G extravasation, and ameliorates neurological deficits. HYDAMTIQ reduces neurological impairment by up to 40% INO-1001 reduces infarct volume by 86%, prevents AIF migration to the nucleus	Cultured human neurons exposed to oxygen–glucose deprivation (OGD)	Olaparib reduces OGD-induced neuronal cell death.	[106,107,108,109]
Multiple sclerosis	A primary demyelination mouse model, induced by a copper chelator cuprizone in weanling mice, results in multi-focal demyelination and loss of oligodendrocytes in the corpus callosum and superior cerebellar peduncle EAE mouse model EAE mouse model	4-hydroxyquinazoline (4HQ) protects against cuprizone-induced demyelination in the brain, prevents weight loss, decreases AIF-mediated cell death. PJ34 suppresses the development of clinical signs of EAE, reduces CNS Inflammation in the spinal cord, and limits BBB disruption. INH2BP delays onset and reduces severity of EAE reducing the expression of pro-inflammatory molecules and immune cells infiltration.	Primary mouse neurons exposed to chondroitin sulfate proteoglycans (CSPGs)	PJ34, 4HQ, or 3AB promote neurite outgrowth	[50,91,92,110]
Traumatic brain injury and Disrupted blood brain barrier	Controlled cortical impact mouse model Controlled cortical impact rat model	PJ-34 administration improves motor functions, reduces AIF release from mitochondria and neuronal loss in cortex and hippocampus. INO-1001 administration suppresses microglia activation	Primary brain microvascular endothelial cells (BMVEC)	5-aminoisoquinoline or Olaparib increases BBB integrity, down-regulate production of pro-inflammatory molecules and decreases human monocyte adhesion, migration through BBB.	[49,107,111]
